# Epitaxial Growth of Nanostructured Li_2_Se on Lithium Metal for All Solid‐State Batteries

**DOI:** 10.1002/advs.202004204

**Published:** 2021-04-09

**Authors:** Hyunjung Park, Jeongheon Kim, Dongsoo Lee, Joonhyeok Park, Seonghan Jo, Jaeik Kim, Taeseup Song, Ungyu Paik

**Affiliations:** ^1^ Department of Materials Science and Engineering Chosun University Gwangju 61452 Korea; ^2^ Department of Energy Engineering Hanyang University Seoul 133‐791 Korea

**Keywords:** all solid‐state batteries, lithium metal, lithium selenide, protective layer, sulfide‐based electrolyte

## Abstract

Lithium is considered to be the ultimate anode material for high energy‐density rechargeable batteries. Recent emerging technologies of all solid‐state batteries based on sulfide‐based electrolytes raise hope for the practical use of lithium, as it is likely to suppress lithium dendrite growth. However, such devices suffer from undesirable side reactions and a degradation of electrochemical performance. In this work, nanostructured Li_2_Se epitaxially grown on Li metal by chemical vapor deposition are investigated as a protective layer. By adjusting reaction time and cooling rate, a morphology of as‐prepared Li_2_Se is controlled, resulting in nanoparticles, nanorods, or nanowalls with a dominant (220) plane parallel to the (110) plane of the Li metal substrate. Uniaxial pressing the layers under a pressure of 50 MPa for a cell preparation transforms more compact and denser. Dual compatibility of the Li_2_Se layers with strong chemical bonds to Li metal and uniform physical contact to a Li_6_PS_5_Csulfide electrolyte prevents undesirable side reactions and enables a homogeneous charge transfer at the interface upon cycling. As a result, a full cell coupled with a LiCoO_2_‐based cathode shows significantly enhanced electrochemical performance and demonstrates the practical use of Li anodes with Li_2_Se layers for all solid‐state battery applications.

## Graphical abstract

Nanostructured Li_2_Se including nanoparticles, nanorods, and nanowalls are epitaxially grown directly on Li metal via chemical vapor deposition for all solid‐state lithium metal batteries. These unique materials have dual compatibility with Li metal and sulfide‐based solid electrolytes and an electrochemical stability, suppressing undesirable side reactions and enabling homogeneous charge distribution as well as long‐term cyclability.

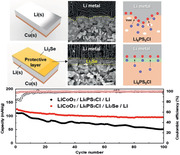

All solid‐state batteries (ASSBs) have recently attracted significant attention due to their enhanced safety compared to conventional LIBs.^[^ ^1^ ^]^ The replacement of flammable liquid organic electrolytes with solid‐state electrolytes (SSEs) can prevent explosions and fires, which makes ASSBs the most promising candidate for application in electric vehicles. Breakthroughs have been made with the development of sulfide‐based electrolytes such as Li_2_S–P_2_S_5_, Li_4‐x_Ge_1‐x_P_x_S_4_, and Li_10_GeP_2_S_12_^[^ ^2^ ^]^ with high Li^+^ ionic conductivity of ≈10^−3^ S cm^−2^ at room temperature.^[^ ^3^ ^]^ However, sulfide‐based solid electrolytes are not stable when in contact with lithium metal, resulting in the decomposition and the formation of various lithium compounds at voltages below 0 V versus Li/Li^+^.^[^ ^4^ ^]^ Lithium alloys have been considered as an alternative to Li metal for ASSB applications. Alloying Li with Al, Ga, In, Sn, or Sb forms a stable interface with sulfide‐based electrolytes and allows for long‐term cycling. Among them, lithium indium (Li/In) is commonly used due to the long plateau at 0.622 V versus Li/Li^+^. However, the utilization of Li/In alloys decreases both the cell voltage and energy density, and the high cost of indium is not suitable for large‐scale applications.

Another main issue is the growth of lithium dendrite. According to previous reports, there are main scenarios for the growth of lithium dendrite in solid‐state batteries.^[^ ^5^ ^]^ First, discontinuous interface contact causes the growth of lithium dendrite. Compared to liquid electrolytes, solid‐state electrolytes normally consist of particles. The physical contact between the SSEs and the Li metal is likely to be a point‐to‐point case. The voids and holes at the interface allow for the dendritic growth of lithium. Second, grain boundary induces lithium dendrite penetration. For most SSEs, polycrystalline are more common. The grain boundaries act as pathways for lithium deposition and propagation in the SSEs. Third, interphase effects on lithium dendrite growth. Most SSEs are thermodynamically unstable against Li metal. The reaction between the SSEs and Li leads to the formation of interphase layers with different properties, leading to an increase in a local current density and a growth of lithium dendrite. In this regard, the introduction of an artificial protective layer could be an effective and straightforward strategy.

Here, we first report on lithium selenide (Li_2_Se) as an artificial solid electrolyte interface for ASSBs. The direct deposition of selenium on Li metal and the subsequent formation of Li_2_Se is a facile and effective method to build a low‐resistance interface between the Li and SSE. The Li_2_Se as a protective layer is thoroughly characterized by various methods, including scanning electron microscopy (SEM), energy dispersive X‐Ray (EDX) spectroscopy, X‐ray diffraction (XRD), and X‐ray photoelectron spectroscopy (XPS) analyses. The advantages of the Li_2_Se protective layer for a stable ASSB operation are demonstrated by direct microscopic observations and electrochemical evaluations.

The strategy of introducing lithium selenide is based on several factors; 1) selenium, the immediate neighbor of sulfur, has similar chemical properties and compatibility with sulfide‐based materials,^[^ ^6^ ^]^ 2) selenium is less reactive toward lithium and more controllable,^[^ ^7^ ^]^ 3) depending on vapor pressure, a small number of atoms and molecules of liquid‐state selenium easily evaporates from the surface in the range of 100–500 ℃ above the melting temperature and below the boiling temperature, and gas‐phase selenium can react with metals, resulting in various metal compounds,^[^ ^8^ ^]^ and 4) one of metal selenides, Li_2_Se, has low electrical conductivity (bandgap: ≈2.997 eV) and could have higher ionic conductivity than Li_2_S (≈10^−5^ S cm^−1^), which is suitable for a protective layer.^[^ ^6^, ^9^ ^]^ **Figure** **1**a shows an illustration of the synthesis procedure of nanostructured Li_2_Se materials with three different structures epitaxially grown on Li metal. First, a Li metal attached onto the copper foil and a selenium power are placed into a quartz tube in a CVD system. During heating at 300 °C under a flow of Ar gas, liquid Li metal reacts with vaporized Se, resulting in the formation of a lithium selenium compound with a stoichiometry of Li_2_Se. After holding for 1min and quenching, Li_2_Se nanoparticles are formed on the surface of the Li metal (Li/Li_2_Se‐NP). In contrast, slow cooling of the specimens leads to the Li_2_Se nanorods and nanowalls (Li/Li_2_Se‐NR and Li/Li_2_Se‐NW, respectively) depending on the holing time for 1min and 10 min, respectively. Due to a poor wettability of a copper substrate toward lithium metal prevents a flow of molten lithium and a morphological change during the CVD process (Figure S1, Supporting Information).^[^ ^10^ ^]^ Top‐view SEM images are displayed in Figure 1b–d. Compared to the bare Li foil surface with bumps and microcracks (Figure S2, Supporting Information), the Li/Li_2_Se‐NP sample consists of nanoparticles with a diameter of ≈100 nm without microcracks (Figure 1b). For the Li/Li_2_Se‐NR, nanorods with a length of ≈500 nm and width of ≈100 nm were observed on the surface (Figure 1c). For the Li/Li_2_Se‐NW sample, nanowalls with a length of ≈500 nm were shown (Figure 1d). Thicknesses of the Li_2_Se layers were found to be around 10, 10, and 15 µm, respectively, before pressing for a full cell assembly (Figure 1e–g). Figure 1h shows XRD patterns. Bare Li shows three distinct peaks (●) that correspond to (110), (200), and (211) planes, respectively. For the Li/Li_2_Se‐NP, Li/Li_2_Se‐NR, and Li/Li_2_Se‐NW samples, new peaks from the Li_2_Se phase (▼) appeared at ≈42° and 50°, while Li metal was recrystallized along the (110) plane. In heteroepitaxy, a crystalline layer or film grow on a crystalline substrate of a different material, and newly formed material has one or more well‐defined orientation with respect to the substrate. In this regard, the dominant (220) plane of the Li_2_Se layer is affected by the (110) plane of the Li metal substrate, which indicates the epitaxial growth of the Li_2_Se on the Li metal. This is highly possible because Li and Li_2_Se possess the same cubic crystal structure (Table S1, Supporting Information), and Se atoms are located at the bridge site between two adjacent Li atoms, as shown in a unit cell of Li_2_Se through <110> projection (Figure 1i). According to the previous report on density functional theory and ab initio molecular dynamics simulations,^[^ ^11^ ^]^ Li_2_S molecules adsorb parallel to the Li (110) plane via strong chemical bonds, and the formation of a Li_2_S film is thermodynamically favorable. In this respect, the formation of Li_2_Se on Li metal could also favor in terms of thermodynamic because the Li_2_Se has the same space group and similar lattice parameters with those of the Li_2_S (Table S1, Supporting Information). To investigate the chemical composition, XPS spectra were obtained after selenium deposition. Bare Li metal (dashed line) has one main peak at ≈56.1 eV that is originated from Li 1s (Figure 1j). After selenium deposition, the spectrum was deconvoluted into main two peaks including an overlap of Li 1s and Se 3d_3/2_ at ≈56.1 eV and Se 3d_5/2_ at ≈54.2 eV, respectively.^[^ ^12^ ^]^ For Se 3p spectra, the Li/ Li_2_Se‐NP sample presents two strong peaks at ≈161.6 and ≈167.5 eV corresponding to Se 3p_3/2_ and Se 3p_1/2_, respectively (Figure 1k). An SEM‐EDX image further confirms the presence of Se on the surface (Figure S3, Supporting Information). Based on the thesis and the characterizations, the proposed growth mechanism for nanostructured Li_2_Se is as follows; Solid lithium metal transforms into a liquid state at the temperature of 300 °C. Selenium vapor adsorbs on the surface of the liquid Li and diffuses inward upon heating, resulting in the formation of a lithium selenide film with a stoichiometry of Li_2_Se. With rapid cooling to room temperature (quenching), Li_2_Se nanoparticles are formed due to a lack of time for grain growth. In contrast, slow cooling leads to the recrystallization of Li metal along the (110) plane (the close‐packed plane of the bcc structure with minimum energy), and grain growth of Li_2_Se subsequently occurs along the (220) plane on the (110) surface of Li metal, resulting in the formation of Li_2_Se nanorods or nanowalls depending on the reaction time.

**Figure 1:**
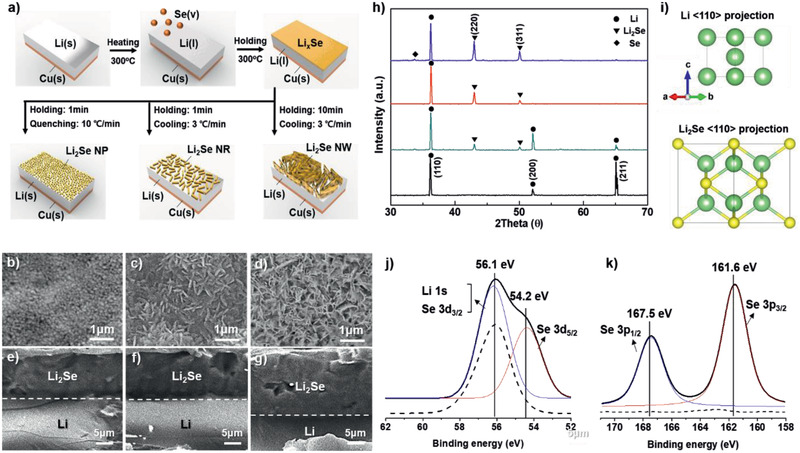
a) Illustration of synthesis procedures for nanostructured Li_2_Se on Li metal by chemical vapor deposition. b–d) Top‐view and e–g) cross‐view SEM images of Li_2_Se‐NP, Li_2_Se‐NR, and Li_2_Se‐NW on Li metal. h) XRD patterns of Li metal (black line), Li/Li_2_Se‐NP (red line), Li/Li_2_Se‐NR (blue line), and Li/Li_2_Se‐NW (pink line). i) Unit cells of Li and Li_2_Se projected along <110> direction. XPS spectra of j) Li 1s/Se 3d and k) Se 3p for Li/Li_2_Se‐NR.

Prior to evaluating electrochemical performance, characterizations of the Li_6_PS_5_Cl electrolyte prepared by high‐energy ball milling were carried out. SEM images showed a secondary particle size in the range of 5–10 µm (Figure S4, Supporting Information). XRD analysis revealed the Li_6_PS_5_Cl particles have high crystallinity with no Li‐bearing secondary phases such as Li_2_S and Li_4_P_2_S_6_. The patterns are well matched with those of previous reports (Figure S5, Supporting Information).^[^ ^13^ ^]^ From EIS data (Figure S6, Supporting Information), the ionic conductivity was calculated to be 2 × 10^−3^ S cm^−1^ at 25 ℃, which is in the range of 10^−4^–10^−3^ S cm^−1^ from previous reports.^[^ ^13^ ^]^ For electrochemical test, uniaxial pressure was applied on the electrodes to reduce contact resistance between the components in the cell (Figure S7, Supporting Information). SEM observation after pressing shows the Li/Li_2_Se‐NP sample has smooth and dense surface layer with no discernable pores (Figure S8a, Supporting Information), and the Li/Li_2_Se‐NR specimen exhibits a dense surface layer consisting of compressed nanorods (Figure S8b, Supporting Information). In contrast, the Li/Li_2_Se‐NW sample became denser due to the pressing and folding of nanowalls, but pores remained between the grains (Figure S8c, Supporting Information). XRD pattern demonstrates that mechanical pressing does not have an appreciable impact on the crystallinity of Li_2_Se despite the morphological change (Figure S9, Supporting Information).

**Figure** **2** shows voltage profiles of four samples upon Li plating/stripping. At a low current density of 0.1 mA cm^−2^, a bare Li cell displayed an average overpotential of ≈25 mV, larger than the ≈8, 9, and ≈10 mV values obtained from the Li/Li_2_Se‐NP, Li/Li_2_Se‐NR, and Li/Li_2_Se‐NW cells, respectively (Figure 2a,d). In this case, all samples exhibited stable operation without noticeable malfunctioning. At a higher current density of 0.2 mA cm^−2^, all samples exhibited a slight increase in overpotential up to ≈25, 10, 12, and ≈20 mV (Figure 2b), respectively, while the overpotential of the Li metal suddenly dropped after 40 cycles and the cell stopped at the 60th cycle, possibly due to an internal short circuit (Figure 2e). Under the condition of 0.5 mA cm^−2^ (Figure 2c,f), all the samples show a significant increase in the overpotentials, and a similar behavior with those under 0.2 mA cm^−2^ was observed. Rate capability was also tested at different current densities (Figure S10, Supporting Information). Three cells with the Li_2_Se protective layers exhibited a gradual augmentation of overpotential as the current densities increased from 0.1 to 0.7 mAh cm^−2^ while the bare Li cell showed the short circuit under 0.3 mAh cm^−2^. As a result, the Li metals with the protective layers possess the enhanced cycle and rate performances than those of the pristine Li metal. On the other hand, the larger overpotential of the Li/Li_2_Se‐NW cell than those of the Li/Li_2_Se‐NP and Li/Li_2_Se‐NR is attributed to the thicker layer of the Li_2_Se protective layer, leading to a longer Li ion pathway. Although there was no a significant difference, the Li/Li_2_Se‐NR exhibited better Li striping/plating property than that of the Li/Li_2_Se‐NP. This might be due to the epitaxial growth of the Li_2_Se nanorods with the dominant (220) plane, which is in accordance with the density functional theory and simulations.^[^ ^8^ ^]^ Hence, the Li/Li_2_Se‐NR sample with the best performance was selected for further characterizations and full cell tests.

**Figure 2:**
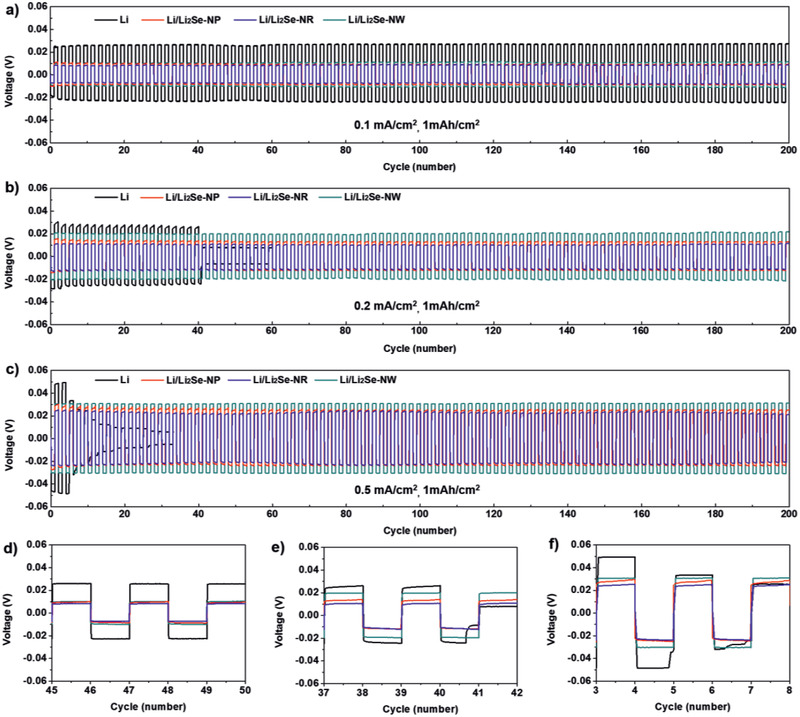
Galvanostatic voltage profiles of symmetric cells of the Li metal (black), the Li/Li_2_Se‐NP (dark cyan), the Li/Li_2_Se‐NR (red), and the Li/Li_2_Se‐NW (blue) with an areal capacity of 1 mAh cm^−2^ at a current density of a,d) 0.1 mA cm^−2^, b,e) 0.2 mA cm^−2^, and e,f) 0.5 mA cm^−2^ over 100 cycles.

To investigate the internal resistance in the cell, EIS spectra of two symmetric cells, Li/Li_6_PS_5_Cl/Li and Li/Li_2_Se‐NR/Li_6_PS_5_Cl/Li_2_Se‐NR/Li were measured. Nyquist plots after 1 and 50 cycles were shown in **Figure** **3**a,b. Equivalent circuits displayed in the inset of the figure identify the major components of resistances: ohmic resistance (*R*_Ω_) from the Li_6_PS_5_Cl electrolyte, interfacial resistance (*R*_1_) from solid electrolyte interface and/or protective layer, charge transfer resistance (*R*_2_) at the Li metal surface, and Warburg impedance (*Z*_w_) as the resistance of mass transfer. Calculated values are summarized in Table S2 (Supporting Information). The *R*_1_ and *R*_2_ of the bare Li cell, as determined from the size of semicircles, were 62 and 91 Ω after 1 cycle and 93 and 124 Ω after 50 cycles, respectively. The overall resistance increased upon subsequent cycling, which may be due to the decomposition of the argyrodite‐type Li_6_PS_5_Cl, the formation of lithium compounds with low ionic conductivity such as Li_2_S, Li_3_P, LiCl, etc.^[^ ^14^ ^]^ In contrast, for the cell with Li_2_Se‐NR, the semicircle corresponding to *R*_1_ (81 Ω) was larger than that of *R*_2_ (65 Ω), which can be attributed to the dense protective Li_2_Se layer. However, the Li_2_Se‐NR shows a much smaller increase in both *R*_1_ (90 Ω) and *R*_2_ (68 Ω) after 50 cycles, resulting in 127 Ω of total resistance. This is lower than the 163 Ω of total resistance for the bare Li cell. Ex situ SEM on cross‐sections shows the Li cell consists of two layers of the Li metal and the Li_6_PS_5_Cl electrolyte (Figure 3c). In contrast, three distinct layers of the Li metal, Li_2_Se‐NR, and Li_6_PS_5_Cl electrolyte for the Li/Li_2_Se‐NR cell were clearly observed (Figure 3d). The thickness of the Li_2_Se‐NR layer decreased from 10 to 5 µm after pressing. It should be noted that the Li cell exhibits a significant change after 50 cycles (Figure 3e). In particular, the Li_6_PS_5_Cl electrolyte lost its original shape and became more porous. According to the previous report on lithium deposition/exfoliation in all solid‐state batteries with sulfide‐based electrolytes,^[^ ^15^ ^]^ lithium deposits in a convex shape like sheets along pores and grain boundaries in the sulfide‐based electrolyte, which can lead to a formation of large cracks and a growth of lithium dendrite. This result agrees with our data although there might be a discrepancy originated from an initial pore size and porosity, operating conditions such as a cell pressure, a current density, a cycle number, *etc*. In this respect, the formation of such a porous structure in the Li_6_PS_5_Cl electrolyte is attributed to a decomposition of the electrolyte and a growth of lithium dendrite during cycling. Furthermore, the uneven interface can cause an inhomogeneous charge distribution, local accumulation of Li metal as shown in Figure 3h, resulting in a potential growth of Li dendrite. Such a scenario has been observed in liquid electrolyte‐based lithium metal batteries.^[^ ^16^ ^]^ The Li/Li_2_Se‐NR cell also shows a transformation, but the interface is flat without noticeable protrusions, and the Li_6_PS_5_Cl layer retained its dense structure without pores (Figure 3f), enabling uniform Li‐ion and electron distribution as well as transfer at the interface (Figure 3i). According to previous report,^[^ ^5^ ^]^ the artificial interface layer should have good compatibility both with Li and SSEs, uniform coverage, ionic conductivity, poor electronic conductivity, and good mechanical strength. In these respects, the Li_2_Se directly grown on the Li metal satisfies most of the requirements for the artificial interface layer. It has compatibility with lithium and sulfide‐based electrolytes due to its similar chemical properties. The Li_2_Se densely cover the whole region of the lithium without microsized cracks, holes, and grain boundaries, which can block the direct physical contact between the SSEs and the Li metal as well as penetration of Li dendrite. Li migration energy of lithium chalcogenides, Li_2_X (X = S, Se and Te), was calculated to be in the range of 0.2–0.3 eV, which indicates they can have lithium ionic conductivity.^[^ ^9a^ ^]^ In addition, the Li_2_S have been studied as a protective layer for Li‐ion batteries, and they successfully prevent the growth of lithium dendrites during cycling, resulting in enhanced electrochemical performances.^[^ ^17^ ^]^ Therefore, our experimental data and previous reports demonstrate that lithium chalcogenides such as Li_2_Se can be a potential candidate for the artificial protective layer.

**Figure 3:**
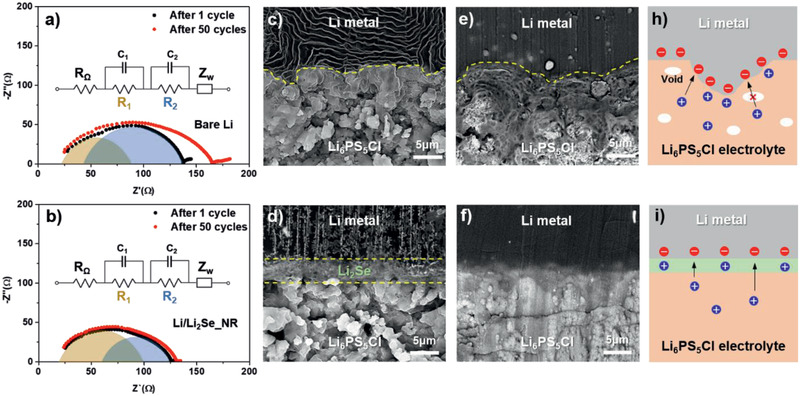
EIS spectra of symmetric cells of a) Li metal and b) Li/Li_2_Se‐NR after 1 and 50 cycles; insets show equivalent circuits of the cell resistance. Cross‐section SEM images of the Li metal and Li/Li_2_Se‐NR cells c–d) before cycle and e–f) after 50 cycles at 0.1 mA cm^−2^. Scheme of charge distribution at the interface for h) the Li metal cell and i) the Li/Li_2_Se‐NR cell.

To explore the practical use of Li_2_Se as a protective layer, full cells employing three different anodes were tested. Specific charge capacities (delithiation) of 135, 148, and 144 mAh g^−1^ with coulombic efficiencies of 87, 86 and 84% were obtained for the cells employing bare Li, Li/In alloy, and Li/Li_2_Se‐NR, respectively, as shown in **Figure** **4**a. The full cell employing bare Li anode showed larger voltage hysteresis (i.e., polarization) than those of the other cells due to the larger cell resistance. Figure 4b displays the rate capability at different current densities. They operated up to a high current density of 1 C‐rate, but the full cell employing the Li/In and the Li/Li_2_Se‐NR anode retained much higher capacity through all rates compared to the full cell employing bare Li anode. The long‐term cyclability at 0.1 C‐rate over 100 cycles is displayed in Figure 4c. While the full cell with the Li anode reached 99% coulombic efficiency after 20 cycles due to undesirable side reaction, the full cell with the Li/In and the Li/Li_2_Se‐NR anode achieved such an efficiency within only 5 cycles. In addition, average efficiencies of three samples were 99.5%, 99.9%, and 99.9% in the range of 20–100 cycles. The capacity retentions of the full cells with the Li, the Li/In, and the Li/Li_2_Se‐NR anodes were 47%, 70%, and 76% after 100 cycles, respectively. In contrast to the continuous capacity fading observed for the Li metal‐based full cell, the full cell employing Li/Li_2_Se‐NR anode exhibited stabilization at ≈100 mAh g^−1^ after subsequent cycles, which is almost equivalent to the cyclability of the full cell with Li/In anode. The capacity decrease in the full cell with Li/Li_2_Se‐NR anode is mainly attributed to degradation of the LiCoO_2_ cathode, while the capacity drop for the full cell with bare Li metal may be due to a synergetic effect on the cathode and anode side. For a comparison, electrochemical performances of all solid‐state batteries based on the artificial interface layer were summarized in Table S3 (Supporting Information).^[^ ^18^ ^]^ It should be noted that a capacity retention is in the range of 75–90% over 100 cycles. A reason for the lower value of this work might be a higher voltage range up to 4.3 V versus Li/Li^+^, which can cause a more electrochemical degradation of the LiCoO_2_.

**Figure 4:**
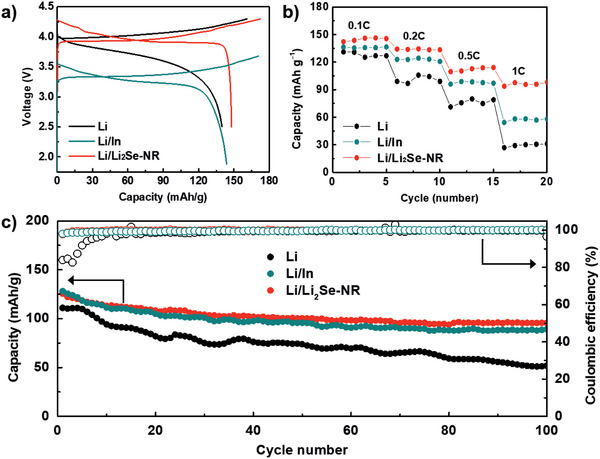
Electrochemical performance of full cells assembled with three different anodes of Li metal, Li/In alloy, and Li/Li_2_Se‐NR. a) Voltage profiles at 0.05 C‐rate. b) Rate capability at 0.05–1 C‐rate. c) Cyclability at 0.1 C‐rate.

In summary, Li_2_Se was successfully introduced as the protective layer for all solid‐state lithium metal batteries. The Li_2_Se was formed directly on the Li metal at a low temperature of 300 °C via CVD method. By controlling the reaction time and cooling conditions, as‐prepared Li_2_Se samples with morphologies consisting of nanoparticles, nanorods, or nanowalls were produced. In symmetric cell tests, the Li/Li_2_Se‐NR electrode showed the smallest overpotential and more stable cycle performance over 140 cycles than those of the Li and Li/Li_2_Se‐NW cells. Consequently, the full cell with Li/Li_2_Se‐NR anode showed enhanced electrochemical performance. The devised strategy of employing an artificial protective layer will be beneficial to the practical use of lithium metal in all solid‐state batteries with sulfide‐based electrolytes.

## Experimental Section

All experimental details are included in the Supporting Information.

## Conflict of Interest

The authors declare no conflict of interest.

## Supporting information

Supporting Information

## Data Availability

The data that support the findings of this study are available from the corresponding author upon reasonable request.
