# Supplementary material for: Epitaxial Growth of Nanostructured Li2Se on Lithium Metal for All Solid‐State Batteries
Source: Adv Sci (Weinh). 2021 Apr 9;8(11):2004204. doi: 10.1002/advs.202004204 (PMC8188223; doi:10.1002/advs.202004204)
Supplement: Supplementary file 1 — Supporting Information [file ADVS-8-2004204-s001.pdf]

WILEY-VCH

© 2021 Wiley-VCH GmbH

ADVANCED SCIENCE
Open Access

Supporting Information

for *Adv. Sci.*, DOI: 10.1002/advs.202004204

Epitaxial Growth of Nanostructured Li_2Se on Lithium Metal for All Solid-State Batteries

Hyunjung Park[‡], Jeongheon Kim[‡], Dongsoo Lee, Joonhyeok Park, Seonghan Jo, Jaeik Kim, Taeseup Song[*], and Ungyu Paik[*]

Supporting Information

Epitaxial Growth of Nanostructured Li_2Se on Lithium Metal for All Solid-State Batteries

Hyunjung Park‡, Jeongheon Kim‡, Dongsoo Lee, Joonhyeok Park, Seonghan Jo, Jaeik Kim, Taeseup Song, and Ungyu Paik**

Experimental Section

Epitaxial growth of nanostructured Li_2Se on Li metal: Lithium foil (T = 500 μm) was attached to a copper current collector via mechanical pressing. Selenium powder (Sigma-Aldrich, -100 mesh, 99.99%) was then placed into an alumina crucible. The crucible and Li/Cu foil were subsequently placed in the center of a quartz tube, and both ends of the tubes were sealed with a Kapton tape. All those processes were conducted in an Ar-filled glove box. The quartz tube with the sample was taken out and quickly connected to gas inlet and outlet parts in a CVD system. Then, the temperature was raised to 300 °C at a rate of 5 °C/min under a 50 sccm flow of Ar gas. For the preparation of Li_2Se nanoparticles, the temperature was maintained at 300 °C for a time no greater than 10 min, at which point the sample was quenched to room temperature by opening the furnace door. For the preparation of Li_2Se nanorods and nanowalls, the temperature was maintained at 300 °C for 1 and 10 min, respectively, followed by cooling to room temperature without opening the furnace door.

Preparation of agyrodite-type Li_6PS_5Cl electrolyte: Li_2S (Sigma-Aldrich, 99.98%), P_2S_5 (Sigma-Aldrich, 99.9%), and LiCl (Sigma-Aldrich, 99%) were used as starting materials. The materials were mixed in a molar ratio of 5:1:2 (Li_2S:P_2S_5:LiCl), and the composite was placed into a zirconia pot (45 mL). Milling was carried out with a planetary ball mill (Pulverisette 7, Fritsch) at 500 rpm for 10 h at room temperature. The obtained mixtures were subsequently heated at 550 °C (ramp rate: 2 °C/min) for 5 h, followed by slow cooling to room temperature.

Measurement of ionic conductivity of the Li_6PS_5Cl electrolyte: 100 mg of as-prepared Li_6PS_5Cl powder was put in a polycarbonate tube (diameter of 10 mm) and pelletized under 300 MPa. A thickness of as-prepared pellet was around 1000 μm. Stainless steel (SUS) disks as a blocking electrode were attached to both sides of the Li_6PS_5Cl pellet. Electrochemical impedance spectrocsopy (EIS) was used to obtain a Nyquist plot of a symmetric SUS/Li_6PS_5Cl/SUS cell by a potentiostat (Wontech, ZIVE BP2) at room temperature. A value of bulk resistance (R_b) was obtained from an intercept of x-axis in the Nyquist plot by simulating and fitting the EIS spectrum (Wontech, Smart manage). Finally, the ionic conductivity was calculated by the equation, $\sigma = d/(R_b \times A)$, where σ is the conductivity, d is the thickness, R_b is the bulk resistance, and A is the area of the electrolyte pellet.

Material Characterization: The morphology of the prepared powders was characterized by field-emission scanning electron microscopy (FE-SEM, JEOL JSM07600F), while element mapping was carried out with an electron-probe microanalyzer (JXA-8100, JEOL). X-ray diffraction patterns of the samples were obtained with an X-ray diffraction analyzer (XRD, Rigaku D/MAX RINT-2000). X-ray photoelectron spectrometry (XPS, VG Microtech ESCA2000) was utilized for chemical composition analysis.

Evaluation of electrochemical properties: For the symmetric cell, 100 mg of Li_6PS_5Cl electrolyte was placed in a polycarbonate tube with a diameter of 10 mm and pressed with a pelletizer under a pressure of 50 MPa. As-prepared anodes including the Li metal and Li/Li_2Se-NR were attached to both sides of the solid electrolyte. The symmetric electrode was subsequently pressed under a pressure of 50 MPa. For the full cell, the cathode material was prepared by mixing $LiNbO_3$-coated $LiCoO_2$ powders (Sigma-Aldrich, 99.8% trace metals basis) and the Li_6PS_5Cl electrolyte in a weight ratio of 70:30 using an agate mortar. The cathode composite powder was then spread on one side of the as-prepared Li_6PS_5Cl electrolyte pellet in a polycarbonate tube and pressed at 300 MPa. Li metal-based anodes were attached to the opposite side of the electrolyte and pressed at 50 MPa. A loading level of the

active material, $LiCoO_2$, was around 7.53 mg cm^{-2}. All processes for the preparation of electrodes and cells were performed in a Ar-filled glovebox. For the symmetric cell test, lithium stripping/plating was carried out at current densities of 0.1, 0.2, and 0.5 mA/cm^2. The full cells were tested over a voltage range of 2.5–4.3V *vs*. Li/Li^+. In the case of the Li/In anode, the cell was evaluated in a voltage range of 1.88–3.68V *vs*. Li-In/Li^+-In. The electrochemical performance of all samples was evaluated with a TOSCAT 3000 battery tester (TOSCAT 3000, Toyo systems, Tokyo Japan).

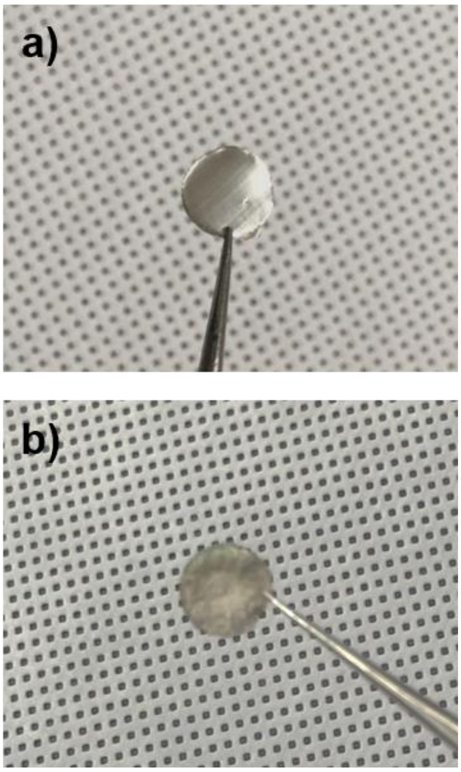

Figure S1. Digital image of round-shaped lithium metal (a) before and (b) after CVD process.

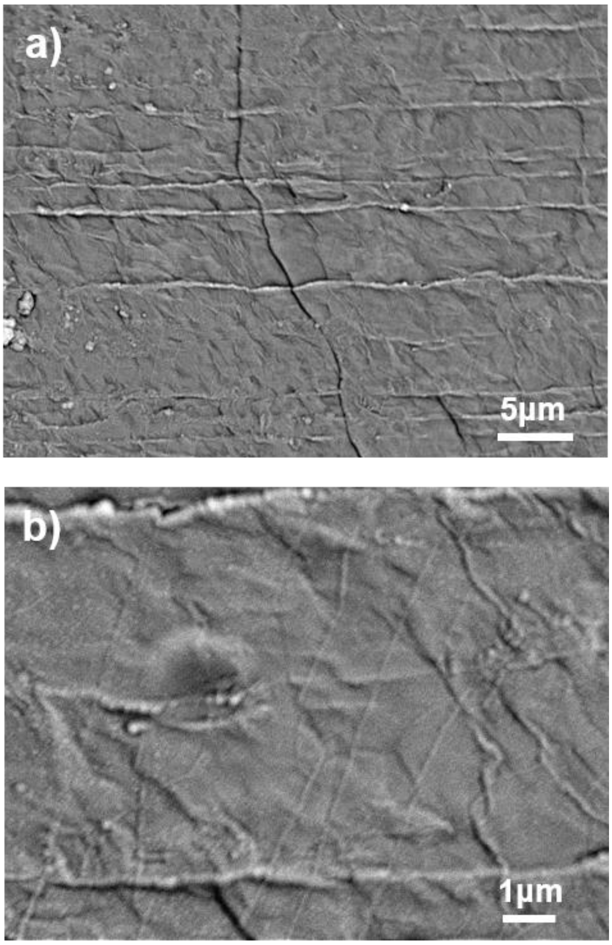

Figure S2. (a) low-magnification and (b) high-magnification SEM images of bare Li metal foil.

Table S1. Crystallographic information of Li and Li_2Se.

Phase	JCPDF	Crystal system	Space group	Lattice parameters		
				a = b = c (Å)	α = β = γ (°)	Volume (Å^3)
Li	00-015-0401	Cubic	Im-3m	3.510	90	43.24
Li_2Se	01-077-2146	Cubic	Fm-3m	6.005	90	216.53
Li_2S	00-023-0369	Cubic	Fm-3m	5.720	90	187.15

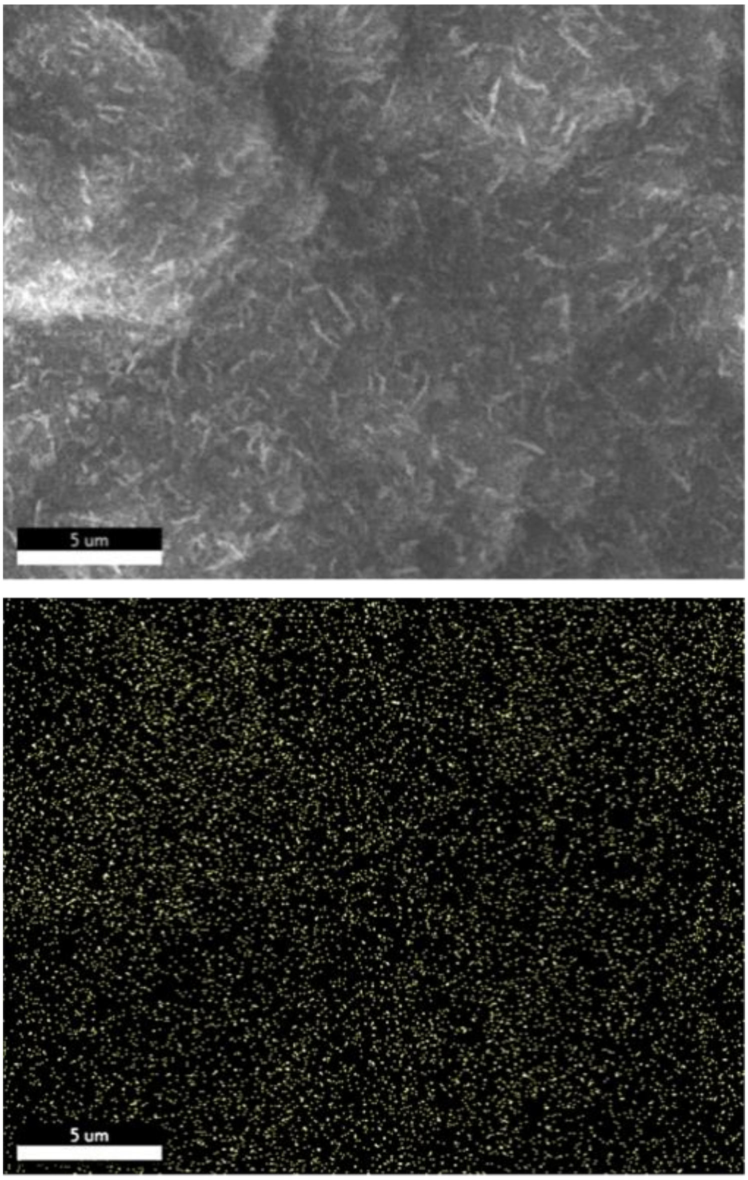

Figure S3. Top-view SEM image of Li metal after selenium deposition and EDX map for Se element.

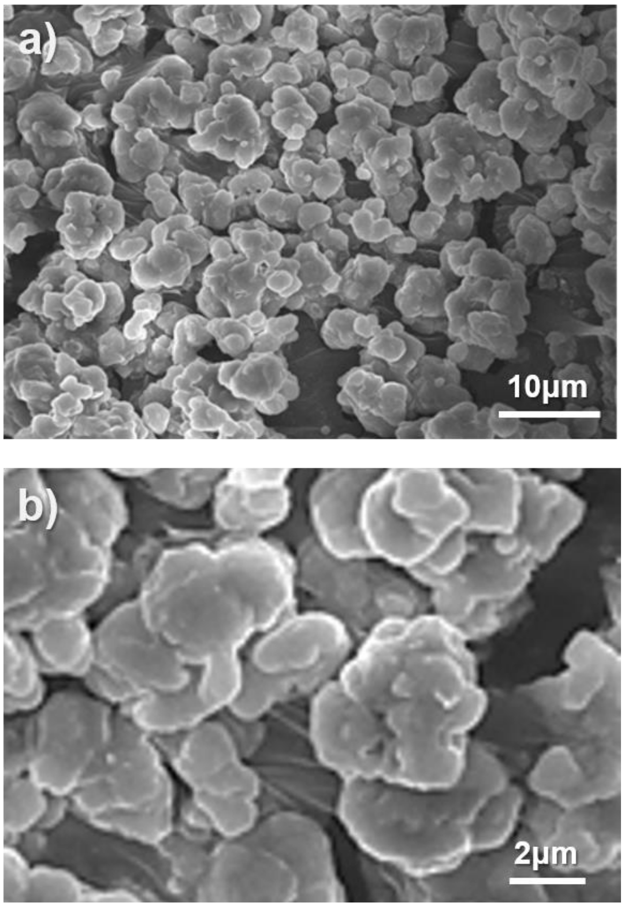

Figure S4. (a) Low- and (b) high-magnification of SEM images of agyrodite Li_6PS_5Cl electrolyte.

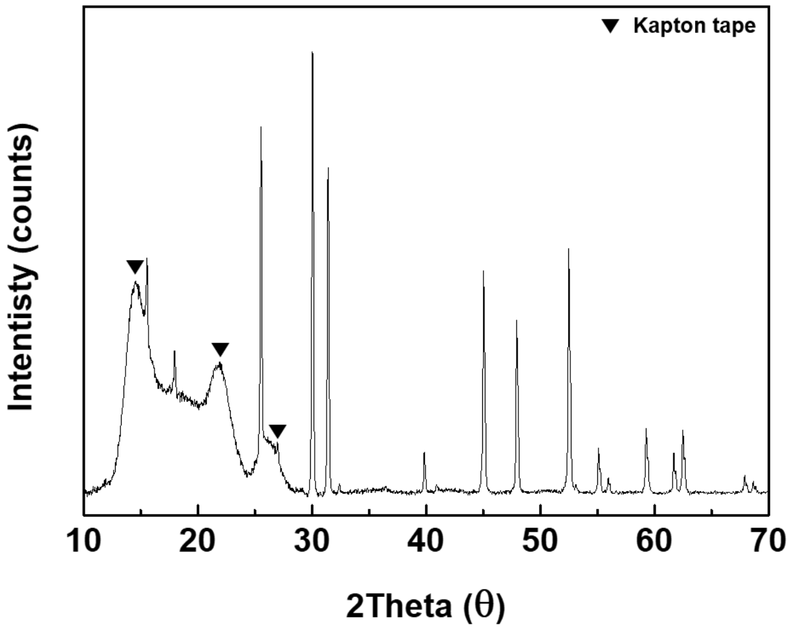

Figure S5. XRD patterns of as-prepared agyrodite Li_6PS_5Cl electrolyte.

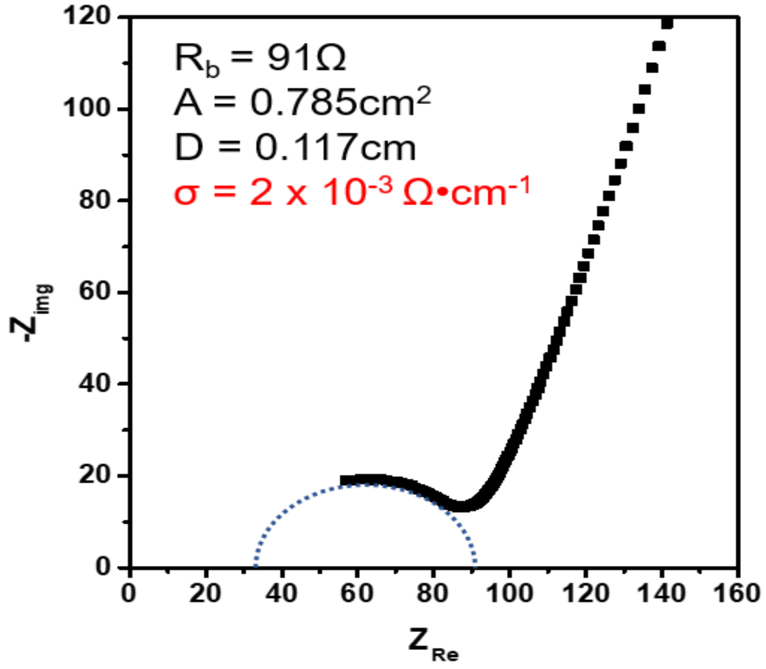

Figure S6. EIS spectra of agyrodite Li_6PS_5Cl electrolyte

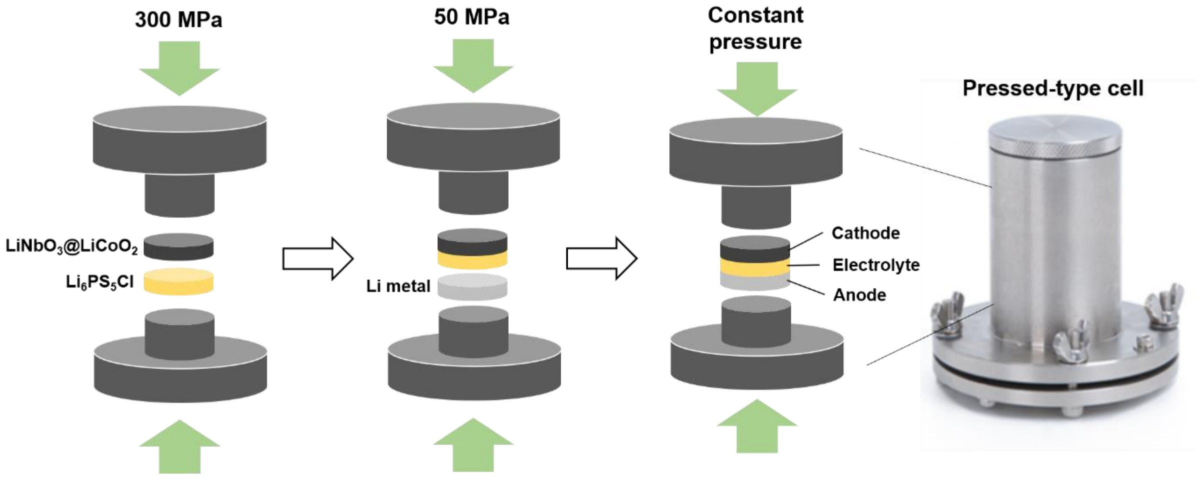

Figure S7. Illustration of the cell assembly and a digital photo of the pressd-type cell.

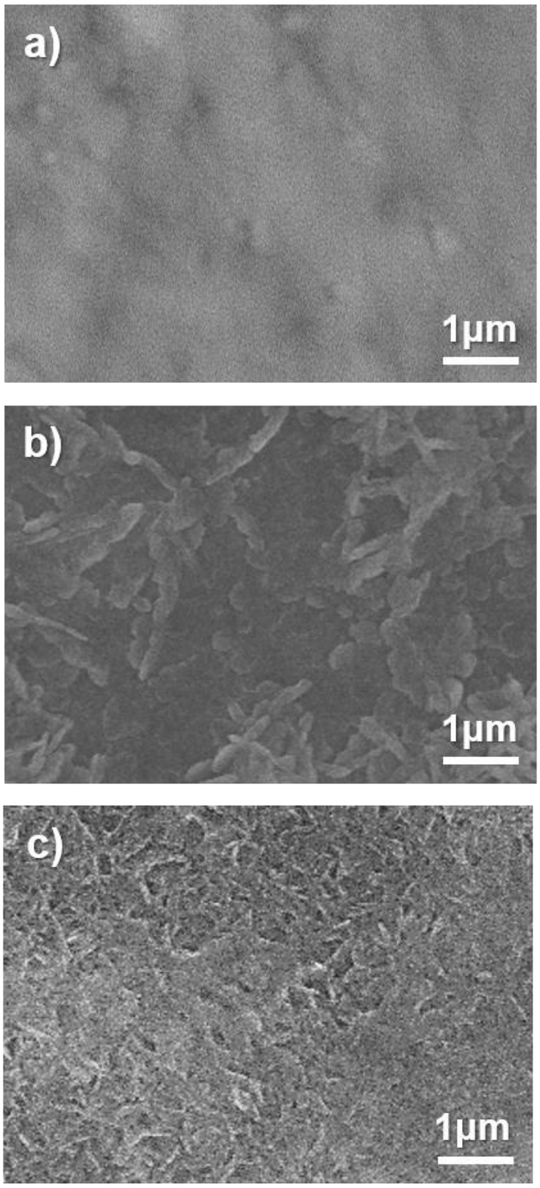

Figure S8. SEM images of (a) Li_2Se nanoparticles, (b) Li_2Se nanorods, and (c) Li_2Se nanowalls after press under 50 MPa.

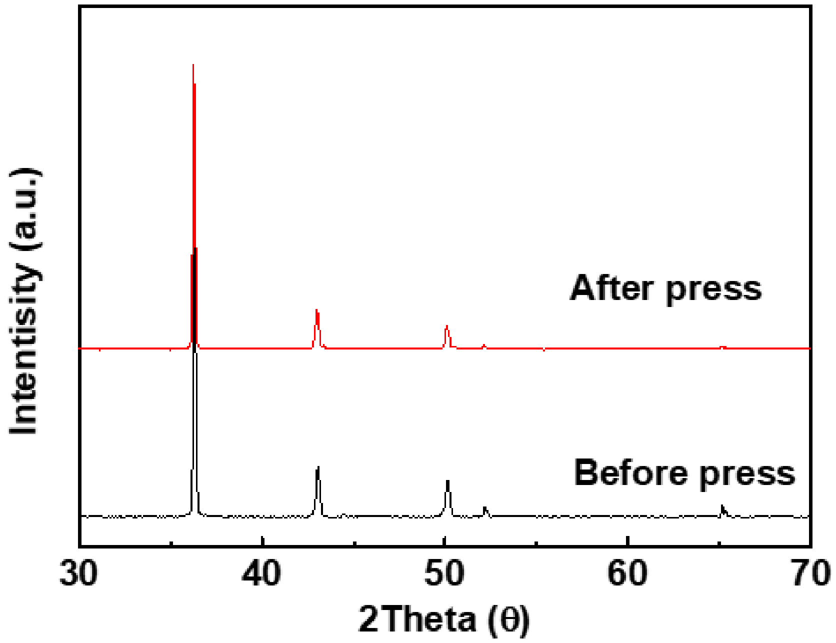

Figure S9. XRD patterns of the Li/Li_2Se-NR before/after press.

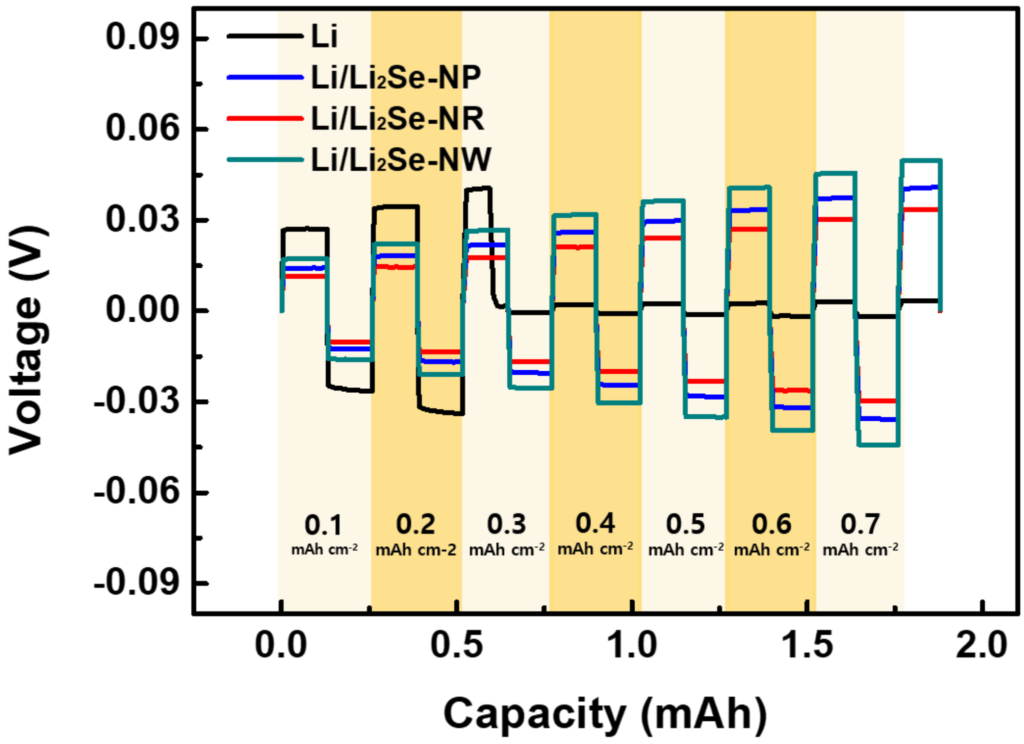

Figure S10. Galvanostatic voltage profiles of symmetric cells of the Li metal (black), the Li/Li_2Se-NP (dark cyan), the Li/Li_2Se-NR (red), and the Li/Li_2Se-NW (blue) at different current densities from 0.1 to 0.7 mAh cm^{-2}.

Table S2. Calculated values of resistance for symmetric cells assembled with Li metal and Li/Li_2Se-NR after 1 and 50 cycles.

Sample		Resistance (Ω)			Total (Ω)
		$R_Ω$	R_1	R_2	
Bare Li	after 1cycle	25	62	93	138
	after 50cycles	18	91	124	163
Li/Li_2Se-NR	after 1cycle	19	81	65	123
	after 50cycles	17	90	68	127

Table S3. Electrochemical performances of all solid-state batteries based on an artificial protective layer for lithium metal.

Cathode	Electrolyte	Protective layer	Anode	Voltage range	Initial discharge capacity	Cycle number	Capacity retention	Reference number
$LiNbO_3$ coated $LiCoO_2$	Li_6PS_5Cl	$Li_6PS_5Cl_{0.3}F_{0.7}$	Li metal	2.8–4.2 V	122 mAh/g at 0.03mA/cm^2	40	95% at 0.13mA/cm^2	15a
$LiZrO_3$ coated $LiCoO_2$	Li_6PS_5Cl	LiPFG, composite of organic PAN/FEC and inorganic Li_3N/LiF	Li metal	2.5–4.2 V	125.7 mAh/g at 0.1C	80	90% at 0.1C	15b
$LiNbO_3$ coated $LiCoO_2$	Li_3PS_4	Li_xSiS_y	Li metal	2.5–4.2 V	126 mAh/g at 0.13mA/cm^2	100	87% at 0.13mA/cm^2	15c
$LiNbO_3$ coated $LiCoO_2$	$Li_7P_3S_{11}$	LiF, lithium fluoride	Li metal	2.5–4.2 V	118.9 mAh/g at 0.1mA/cm^2	100	81.4% at 0.1mA/cm^2	15d
$LiNbO_3$ coated $LiCoO_2$	$Li_7P_3S_{11}$	LiI, lithium iodide	Li metal	2.5–4.2 V	~115 mAh/g at 0.1mA/cm^2	100	77.5% at 0.1mA/cm^2	15d
$LiNbO_3$ coated $LiCoO_2$	Li_6PS_5Cl	Li_2Se, lithium selenide	Li metal	2.5–4.3 V	~144 mAh/g at 0.1mA/cm^2	100	76% at 0.1C	This work
